# Short-Term Outcomes of Surgery for Graves’ Disease in Germany

**DOI:** 10.3390/jcm9124014

**Published:** 2020-12-11

**Authors:** Elisabeth Maurer, Christian Vorländer, Andreas Zielke, Cornelia Dotzenrath, Moritz von Frankenberg, Hinrich Köhler, Kerstin Lorenz, Theresia Weber, Joachim Jähne, Antonia Hammer, Knut A. Böttcher, Katharina Schwarz, Carsten Klinger, Heinz J. Buhr, Detlef K. Bartsch

**Affiliations:** 1 Department of Visceral, Thoracic and Vascular Surgery, Philipps University Marburg Baldingerstrasse, 35043 Marburg, Germany; bartsch@med.uni-marburg.de; 2 Department of Endocrine Surgery, Bürgerhospital Frankfurt/Main, 60318 Frankfurt am Main, Germany; c.vorlaender@buergerhospital-ffm.de; 3 Department of Endocrine Surgery, Diakonie-Klinikum Stuttgart, 70176 Stuttgart, Germany; andreas.zielke@diak-stuttgart.de; 4 Department of Endocrine Surgery, Helios Universityhospital Wuppertal, 42283 Wuppertal, Germany; cornelia.dotzenrath@helios-gesundheit.de; 5 Department of Surgery, Hospital Salem-Heidelberg, 69121 Heidelberg, Germany; Moritz.von.Frankenberg@med.uni-heidelberg.de; 6 Department of General Surgery, Herzogin Elisabeth Hospital Braunschweig, 38124 Braunschweig, Germany; h.koehler@heh-bs.de; 7 Department of Visceral, Vascular and Endocrine Surgery, University Medical Center Halle, 06120 Halle, Germany; kerstin.lorenz@uk-halle.de; 8 Department of Endocrine Surgery, Katholisches Klinikum Mainz, 55131 Mainz, Germany; t-weber@kkmainz.de; 9 Department of General and Visceral Surgery, Diakovere Henriettenstift Hannover, 30171 Hannover, Germany; joachim.jaehne@diakovere.de; 10 Department of Endocrine Surgery, DKD Helios Clinic Wiesbaden, 65191 Wiesbaden, Germany; antonia.hammer@helios-gesundheit.de; 11 Department of General and Visceral Surgery, Diakonissen Hospital Mannheim, 68163 Mannheim, Germany; knut.boettcher@diakonissen.de; 12 Department of Endocrine Surgery, Lukas Hospital GmbH Neuss, 41464 Neuss, Germany; kschwarz@lukasneuss.de; 13 German Society of General and Visceral Surgery, 10117 Berlin, Germany; carsten.klinger@dgav.de (C.K.); hbuhr@dgav.de (H.J.B.)

**Keywords:** Graves’ disease, thyroidectomy, hypoparathyroidism, parathyroid glands, neuromonitoring

## Abstract

Background: Surgical treatment of Graves’ disease (GD) has a potentially increased incidence of postoperative hypoparathyroidism, recurrent laryngeal nerve palsy (RLNP) and bleeding. The aim of this study was to evaluate the current extent of surgery for the treatment of GD and its safety as a short-term outcome. Methods: Patients who underwent thyroid resection for GD were identified from the prospective StuDoQ/Thyroid registry. Patient data were retrospectively analyzed regarding demographics, surgical procedures and perioperative outcomes. Statistics were performed with Student’s *t*-test or Fisher’s exact test and multivariate Cox regression analysis. The level of statistical significance was set at *p* < 0.05. Results: A total of 1808 patients with GD with a median age of 44 (range 14–85) years were enrolled in a 25-month period by 78 departments, of which 35.7% (*n* = 645) had an endocrine orbitopathy and 0.1% (*n* = 6) had thyrotoxic crisis. Conventional open surgery was used in 98.6% of cases and minimally invasive or remote-access approaches were used in 1.4%. Total thyroidectomy was performed in 93.4% of cases (*n* = 1688). Intraoperative neuromonitoring (IONM) was used in 98.9% (*n* = 1789) of procedures. In 98.3% (*n* = 1777) at least one parathyroid gland was visualized and in 20.7% (*n* = 375) parathyroids were autografted. The rates of unilateral and bilateral transient RLNP were 3.9% (*n* = 134/3429 nerves at risk) and 0.1% (*n* = 4/3429 NAR). The rates of transient RLNP tended to be higher when intermittent IONM was used compared to continuous IONM (4.1% vs. 3.4%, *p* < 0.059). The rate of transient postoperative hypoparathyroidism was overall 29% (*n* = 525/1808). Multivariate analysis revealed fewer than 300 thyroid resections and fewer than 15 thyroid resections for GD per year, male sex, BMI > 30, autotransplantation of parathyroid glands and previous bilateral thyroid surgery as independent risk factors for postoperative temporary hypoparathyroidism. Reoperations for bleeding (1.3%) were rare. Conclusion: Total thyroidectomy with IONM is safe and currently the most common surgical therapy for GD in Germany. Postoperative hypoparathyroidism is the major complication which should be focused on.

## 1. Introduction

Graves’ disease (GD) is an autoimmune disorder caused by the production of auto-thyrotropin receptor antibodies against the thyroid-stimulating hormone (TSH) receptor [1]. GD is the most common cause of endogenous hyperthyroidism and may cause various symptoms and a considerable morbidity with increased risk of cardiovascular adverse events. Approximately 3% of women and 0.5% of men will develop GD during their lifetime [2]. Five per cent of patients with GD develop moderate to severe Graves’ ophthalmopathy [1]. For the definitive treatment of GD, two main treatment options currently exist, either radioiodine radiation treatment or thyroid surgery [2,3,4,5]. Furthermore, extended therapy with antithyroid drugs may be included. The primary objective of the operation should be the definitive control of hyperthyroidism, ideally without causing complications. The extent of thyroid surgery for GD is not precisely defined by current guidelines [3,6]. Total thyroidectomy as surgical treatment for GD is considered to be a fast, consistent and permanent treatment to restore and sustain euthyroidism [7,8] and a postoperative hormonal therapy is necessary. Total thyroidectomy is considered to be the preferred surgical treatment for GD in a variety of clinical scenarios namely when involving nodules, suspicions of malignancy, severe endocrine orbitopathy (EO), large goiters or, in the case of pregnancy and GD, hyperthyroidism [9,10]. Different kinds of subtotal resections were the preferred surgical procedures for GD until approximately the 21st century [9] and three former RCTs reported lower rates of hypoparathyroidism after bilateral subtotal thyroidectomy than after total thyroidectomy [11,12,13]. A very recent randomized controlled multicenter trial comparing total and near-total thyroidectomy identified total thyroidectomy as preferred surgical procedure for the treatment of GD with regard to transient and permanent hypoparathyroidism, transient recurrent laryngeal nerve palsy (RLNP), and short-term recurrent hyperthyroidism [14]. In addition, a higher risk of inadvertently removing parathyroid glands when performing a bilateral near-total resection was reported [14]. The current German S2k guideline and the guideline of the American Thyroid Association still recommend both total and near-total resections if surgery is chosen as the definitive therapy for GD [3,6].

The major complication of thyroid resection for GD is postoperative hypoparathyroidism with transient and permanent rates between 18.0–20.0% and 3.0–7.0% [14,15,16,17], followed by postoperative bleeding and recurrent laryngeal nerve palsy (RLNP). The underlying causes are complex. Dissection techniques, especially the extent of resection, seem to be more influential than inadvertent parathyroidectomy [18,19].

The aim of this study was to evaluate the current surgical strategy for the treatment of GD in Germany and its short-term outcomes.

## 2. Materials and Methods

The StuDoQ/Thyroid Registry of the German Society of General and Visceral Surgery (DGAV) was designed as a prospective, multicenter database with web-based data entry, has been maintained since April 2017 and was developed in close cooperation with the board of the German Society of Endocrine Surgeons (CAEK). Relevant outcome and quality indicators were selected on the basis of the German S2k guidelines [6,20] for the surgical treatment of benign and malignant goiters, the certification guidelines for competence centers for thyroid/parathyroid surgery of the DGAV (www.dgav.de), and the EUROCRINE registry (https://eurocrine.eu). A total of 52 parameters were defined by a browser-based electronic case report form (eCRF). Data from participating centers were entered in pseudonymized form at the institutional level without an over-institutional identifier in the prospective StuDoQ/Thyroid registry. The data were immediately subjected to an automatic plausibility check with automatic error indication.

All participating institutions had to approve the correct number of thyroid resections entered in the registry by a written confirmation of the hospital’s administration. In addition, the completeness and correctness of the data was checked as part of the regular certification audits for the certified competence and reference thyroid centers of the DGAV, and by external monitoring since 2019.

All enrolled patients signed an informed consent for data collection and approval for data safety management was obtained from the TMF (Technologie und Methodenplattform für die vernetzte medizinische Forschung).

For the present study, all patients who underwent thyroid resections for Graves’ disease between 1 April 2017 and 30 April 2019 were retrieved from the StuDoQ/Thyroid registry. The diagnosis Graves’ disease was preoperatively based on clinical symptoms of hyperthyroidism, an existing EO, laboratory thyroid function tests as thyroid stimulating hormone (TSH), free triiodothyronine (fT3), thyroxine (fT4) and TSH receptor antibodies, with respect to concomitant substitution or thyrostatic medications to achieve euthyroidism. Patients were considered hyperthyroid with preoperative TSH levels <0.1 mU/L or low TSH levels (0.02–0.34 mU/L). GD was postoperatively confirmed by the pathological analysis. The indications for operation as definitive treatment of GD were according to the national S2k-guideline “operative treatment of benign thyroid diseases” [6] thyroid growth, EO, suspected malignancy, severe side effects or intolerance of antithyroid medication, treatment-resistant or severe hyperthyroidism or refusal of radioiodine therapy by the patient. Data were extracted in anonymized form and retrospectively analyzed with respect to demographics, indication for surgery, type of surgical procedure and perioperative outcome.

The postoperative function of the recurrent laryngeal nerve was determined by laryngoscopy within the first 5 postoperative days by an independent surgeon or head and neck specialist as required in the national S2k-guideline “operative treatment of benign thyroid diseases” [6]. Vocal cord immobility was defined as palsy. Impaired, but notable vocal cord movement was defined as incomplete palsy. Both conditions were documented as dysfunction of the recurrent laryngeal nerve (RLNP, recurrent laryngeal nerve palsy). The palsy rate was calculated per nerves at risk (NAR). The nerve dysfunction was defined as transient if it completely recovered within 6 months and permanent if it persisted after 6 months. Postoperative hypoparathyroidism was defined as a serum level of intact parathormone <15 pg/mL (normal range 15–65) and serum calcium <2.1 mmol/L (normal range 2.1–2.6) with the necessity of calcium substitution. All three conditions had to be fulfilled for diagnosis of hypoparathyroidism. A substitution therapy was only initiated after diagnoses of hypoparathyroidism postoperatively. The postoperative hypoparathyroidism was defined as transient if it completely recovered within 6 months, after which it was considered permanent.

### Statistical Analysis

Continuous variables are presented as means (standard deviation) and categorical variables as proportions. Quantitative variables were compared using the Student’s *t*-test and qualitative variables (e.g., gender) using the chi-square test or Fisher’s exact test as appropriate. The effect of single risk factors on hypoparathyroidism was explored in bivariate logistic regressions. A multivariate logistic regression model was analyzed using stepwise regression.

All reported probability values (*p*-values) are based on two sided tests; the level of statistical significance was set at *p* < 0.05. Analyses were performed using SPSS 23.0 (IBM, Chicago, IL, USA, 2017).

## 3. Results

A total of 27.395 patients were entered into the StuDoQ/Thyroid registry by 90 institutions within 25 months. Of those, 1808 (6.6%) patients had Graves’ disease (GD), who were recruited by 78 (87%) surgical departments. The data of these patients, who were all discharged until April 30th, 2019, were analyzed. A total of 79% (*n* = 1429) of patients with GD were operated in hospitals with more than 300 thyroid resections per year; only 4.9% (*n* = 88) of patients were operated on in hospitals with 100 or fewer thyroid resections per year. A total of 11 institutions enrolled more than 50 patients, 7 institutions 31–50 patients and 9 institutions 21–30 patients with GD, whereas the remaining 51 institutions operated on 20 or fewer patients with GD during the recruitment period (Table 1).

A total of 81.7% (*n* = 1478) of women and 18.3% (*n* = 330) of men with a median age of 44 (range 14–85) years underwent thyroid resection for GD. Only 2.4% (*n* = 43) of patients were younger than 18 years and 3.5% (*n* = 64) patients were older than 70 years. Furthermore, 7.2% (*n* =131) of patients (110 women, 21 men) were overweight with a body mass index > 35 (Table 2). A total of 10.7% (*n* = 193) of patients had an increased perioperative risk with an ASA (American Society of Anesthesiologists) score ≥ 3. A total of 35.7% (*n* = 645) of patients had endocrine orbitopathy and 55.3% (*n* = 999) of patients had biochemical hyperthyroidism (TSH < 0.01 mU/L or low TSH 0.02–0.34 mU/L) at the time of surgery. A total of 1536 of 1808 patients (84.9%) took preoperative antithyroid drugs. Furthermore, 99.7% (*n* = 1802) of patients underwent elective surgery and 0.3% (*n* = 6) had emergency surgery for thyrotoxic crisis. A total of 96.2% (*n* = 1740) of patients had initial surgery and 3.7% (*n* = 66) patients had cervical reoperations. Another two patients (0.1%) underwent radiofrequency ablation of GD prior to surgery. Preoperative laryngoscopy for the assessment of vocal cord function was performed in 98.5% (*n* = 1780) patients. Normal results were obtained in 98.1% (*n* = 1774) of patients and 0.3% (*n* = 6) of patients showed a unilateral palsy of the recurrent laryngeal nerve (RLN). Patient characteristics are summarized in Table 2.

The vast majority of patients underwent total thyroidectomy (92.3%, *n* = 1668), whereas bilateral subtotal resections (0.6%, *n* = 10) and Hartley-Dunhill procedures (0.8%, *n* = 14) were an exception (Table 3). Furthermore, 5.4% (*n* = 98) of the procedures were hemithyroidectomies, mainly as reoperations for either previous neck surgery or as incomplete resections because of suspected RLN damage after resection of the first side (Table 3). The median operative time was 106 min, ranging from 32–358 min. In 98.6% (*n* = 1782) of patients, conventional open surgery was performed, whereas 1.3% (*n* = 24) had a minimally invasive video-assisted thyroidectomy (MIVAT), and 0.1% (*n* = 2) of patients had a robotic-assisted transaxillary thyroidectomy. Other endoscopic or robotic-assisted remote-access approaches like BABA (bilateral axillo-breast approach) were not used. Only 0.05% (*n* = 1) of patients required a partial sternotomy for the resection of GD. In 5.4% (*n* = 97) patients any type of selective or systematic lymphadenectomy was performed, which showed no influence on postoperative hypoparathyroidism (*p* = 0.63) or recurrent laryngeal nerve palsy (*p* = 0.58).

Visualization of the recurrent laryngeal nerve (RLN) was performed in 99.8% (*n* = 1804) of patients. Intraoperative neuromonitoring (IONM) of the RLN was used in 98.9% (*n* = 1789), and intermittent IONM (77.0%, *n* = 1393) was significantly more often used than continuous IONM (21.9%, *n* = 396, *p* < 0.0001). The rate of transient RLN palsy (per NAR) after use of continuous IONM was 3.4% compared to 4.1% when intermittent IONM was used (*p* = 0.059, Table 4). IONM of the superior laryngeal nerve, however, was only performed in 11.7% (*n* = 211) of patients.

In 98.3% (*n* = 1777) of patients at least one parathyroid gland was identified intraoperatively (Table 5); in the majority of patients (96.6%, *n* = 1747) two or more parathyroids were visualized. Autotransplantation of parathyroid glands was undertaken in 20.7% (*n* = 375) of patients. In the vast majority of these patients (87.2%, *n* = 327), two parathyroid glands were autotransplanted.

The median specimen weight was 24 (range 8–242) grams, whereby 4.6% (*n* = 83) weighed less than 10 g and 4.9% (*n* = 88) more than 100 g. Histopathological examination confirmed GD in all patients; one (0.05%) patient showed, in addition to GD, a papillary carcinoma.

Postoperative laryngoscopy was performed in 97.2% (3332 of 3429 NAR) of nerves at risk (NAR). This revealed a vocal cord dysfunction in 4.0% (138 of 3332 NAR), with unilateral dysfunction in 3.9% (*n* = 134 NAR) and bilateral dysfunction in 0.1% (*n* = 4 NAR, Table 4). At the evaluation date, 122 of the 138 NAR with early postoperative dysfunction should have had the 6-month follow-up laryngoscopy. This result was documented for 80% (*n* = 98/122) NAR, and 40% (*n* = 40) of these nerves showed a permanent unilateral palsy, whereas no bilateral dysfunction was present. Based on a follow-up rate of 80%, one can extrapolate a rate of permanent RLNP of about 1.5% per NAR (50/3332).

Postoperative hypocalcemia with the need for calcium substitution was present at discharge in 29% (*n* = 525/1808) of patients; in 2.3% (*n* = 42) of patients even intravenous calcium substitution was supplemented. A total of 354 (67.4%) patients with postoperative hypoparathyroidism (*n* = 525) received simultaneous vitamin D at discharge. The rate of early postoperative hypoparathyroidism was independent of the number of parathyroid glands visualized intraoperatively (Table 5). However, the rate of early postoperative hypoparathyroidism was significantly higher in patients who underwent parathyroid gland autotransplantation compared to patients who had no autograft (43.5% vs. 25.2%, *p* < 0.0001). The rate was highest after autotransplantation of more than two parathyroid glands (64.4%, Table 5). A 6-month follow-up examination was documented at the evaluation date for 309 of 525 (58.8%) patients with postoperative hypoparathyroidism. Of those, 33 showed a persistent hypoparathyroidism. If one assumes none or all of the patients with incomplete follow-up had a permanent hypoparathyroidism, the rate would be between 1.8 and 13.8%. Most likely a projected rate of permanent hypoparathyroidism of 2.9% can be assumed as 33 of 309 patients is a proportion of about 10%.

The overall case number of thyroid resections, case number of thyroid resections for GD, sex, age, BMI, preoperative TSH and PTH level, previous neck surgery, surgical method, autotransplantation of parathyroid glands, lymphadenectomy and thyroid volume were analyzed as potential risk factors for postoperative hypoparathyroidism. A center volume of more than 300 thyroid resections per year (*p* < 0.0005), center volume of more than 30 thyroid resections for GD during the study period (*p* < 0.0005), sex (*p* < 0.0005), age (*p* < 0.0005), BMI (*p* = 0.019), autotransplantation of parathyroid glands (*p* < 0.0005) and previous bilateral thyroid surgery (*p* = 0.028) showed a statistically significant influence on postoperative hypoparathyroidism in the univariate logistic regression, which was confirmed in the multivariate Cox regression analysis (Table 6). Surgical method, previous neck surgery overall, lymphadenectomy, thyroid volume and preoperative TSH and PTH levels, however, showed no significant influence on postoperative hypoparathyroidism.

Reoperations for complications had to be performed in 1.6% (*n* = 29) of patients. These included postoperative bleeding in 1.3% (*n* = 23), wound infection in 0.2% (*n* = 3) and other reasons such as lymphatic fistula in 0.2% (*n* = 3), respectively. Another 14 (0.8%) patients underwent complete of thyroidectomy 2–4 days after the initial operation, since the first procedure was terminated as hemithyroidectomy due to suspicion of RLNP by IONM, which was not confirmed during postoperative laryngoscopy.

## 4. Discussion

The presented data of 1808 patients who underwent surgery for GD are the largest European series ever collected within 2 years. A total of 78 institutions who performed thyroid surgery for GD participated in the StuDoQ/Thyroid database. Almost 80% of patients with GD were operated in 28 high-volume centers with over 300 operations per year. Thus, there is a clear trend towards centralization of surgery for GD nowadays in Germany [21]. A volume-outcome analysis was performed and showed that there is a lower risk for complications when patients with GD were operated on in high-volume centers. A caseload of more than 301 thyroid resections per year and more than 30 resections for GD during the study period of 25 months, roughly equal to 15 resections per year, decreased the risk of postoperative hypoparathyroidism significantly (Table 6). A previous nationwide analysis from the United States with 11.205 patients with GD operated on between 2006 and 2011 also confirmed a lower risk of postoperative complications when treated in high volume centers [22].

Numerous reports over the last years suggest that minimally invasive video-assisted operations (MIVAT [23]) or remote access robotic procedures (bilateral-axillo-breast approach [24], transaxillary approach [25,26]) for the surgical treatment of GD are accepted and frequently used throughout the thyroid community. In Germany, however, these procedures do not play a role in patients with GD, since only 1.3 resp. 0.1% of all procedures in the present study were performed with minimally invasive or remote access approaches.

In the present study, total thyroidectomy was by far the most frequently performed procedure (93.4%) to treat GD, whereas bilateral subtotal resections (0.6%) and Hartley-Dunhill procedures (0.8%) were rarely used. This trend has already been observed in an earlier European questionnaire study covering the years 2010 to 2012 in 2488 patients with GD [16]. This study reported a total thyroidectomy rate for GD of about 90% and a rate of any bilateral subtotal resections below 5%. Although current American, European and German guidelines [3,6,27,28] do not give definite recommendations regarding the extent of thyroid resection for GD, total thyroidectomy is the preferred surgical treatment of choice in the Western world during the last decade.

Total thyroidectomy is regarded as an effective treatment of GD with definitive control of hyperthyroidism and its symptoms [5,29]. Subtotal resection for GD is considered as surgical strategy with a lower rate of postoperative hypoparathyroidism, but a higher risk of persistence or recurrence of disease [30,31]. Five RCTs since the 90 s with a total of 879 patients deal with the extent of thyroid resection for sufficient treatment of GD regarding postoperative complications [11,12,13,14,32]. Different types of uni- or bilateral sub/near-total resections were compared to total thyroidectomy. The overall incidence of postoperative transient hypoparathyroidism varied from 14.7 to 25.1% of patients [11,12,13,14,32]. A meta-analysis of Feroci et al. based on 3 RCTs and 11 retrospective studies reported an overall rate of temporary hypoparathyroidism of 18.6% in 2025 patients [17]. The present study showed an incidence of temporary hypoparathyroidism of 29%. This relatively high prevalence might be explained by the over 90% rate of total thyroidectomies, whereas previous studies comprised more than 50% subtotal resections. However, the presented data are in line with a recent German multicenter RCT, which was conducted in high-volume centers and provided clearly defined parameters. The rate of transient postoperative hypoparathyroidism was 21% after total thyroidectomy [14].

Transient postoperative hypoparathyroidism is caused by transient reduced vascularization or even accidental removal of parathyroid glands. Thus, guidelines recommend the identification and protection of at least one parathyroid gland during surgery for GD [6]. In the present study, in nearly all cases (98.3%) at least one parathyroid gland and in 96.6% of patients two or more glands were identified. This is in line with a previous Swedish multicenter study with 1.157 operations for GD that reported a 94.3% rate of identification of two or more parathyroid glands [33]. In the present study, the identification of no parathyroid gland, as well as identification of any parathyroid gland, did not correlate with transient postoperative hypoparathyroidism (see Table 5). In the above-mentioned Swedish study, the number of identified parathyroid glands was also no risk factor for postoperative hypocalcemia [33]. The rate of parathyroid gland autotransplantation in the present study was 20.7% (375/1808), which is in the range of 12.1 and 35.1% in previously reported larger retrospective studies [33,34] and a little lower than the 26% rate in the TONIG RCT [14]. Surprisingly, in the majority of operations with autotransplantation, not only one but two parathyroid glands were autotransplanted (87.2%, *n* = 327). In the present study, parathyroid autotransplantation was associated with higher risk of postoperative transient hypoparathyroidism (25.2 vs. 43.5%, *p* < 0.0005), which was highest after autotransplantation of two or more parathyroid glands. Former retrospective analyses of thyroid surgery confirmed parathyroid autotransplantation as an independent risk factor for postoperative transient hypoparathyroidism in patients undergoing thyroid surgery for autoimmune thyroid disease [35,36,37,38]. In contrast, older small-scale retrospective studies, such as Karakas et al., showed no influence of parathyroid autotransplantation on postoperative transient hypoparathyroidism after surgery for GD [39]. Another reason for the occurrence of transient postoperative hypoparathyroidism is inadvertent removal of parathyroid glands [14,40,41,42]. The StuDoQ/Thyroid registry contains no valid information about inadvertently removed parathyroid glands, so we could not analyze our data regarding this issue.

One serious complication after thyroid surgery for GD is probably permanent postoperative hypoparathyroidism. In the present study, permanent postoperative hypoparathyroidism was extrapolated in approximately 2.9% of patients. A European questionnaire study of 1141 patients with GD reported a rate of permanent hypoparathyroidism of 2.8% [16]. The most recent German multicenter randomized controlled TONIG trial showed an overall rate of permanent hypoparathyroidism of 3.8% after thyroid surgery for GD [14]. This is approximately 2–3 times higher than after thyroidectomy or bilateral subtotal resection for nodular goiter or low risk thyroid cancer. The reasons for this fact need to be clarified. According to the aforementioned data, permanent postoperative hypoparathyroidism caused by either devascularization or accidental removal of parathyroid glands is nowadays the most serious major complication of surgery for GD, since it requires lifelong medical treatment. Thus, in the future parathyroid preservation needs to be addressed as a main concern in thyroid surgery for GD. New promising techniques of intraoperative identification and localization of parathyroid glands such as indocyanine green angiography [43,44] and autofluorescence spectroscopy [45,46,47] might reduce the risk of permanent hypoparathyroidism.

Patient-specific independent risk factors for postoperative temporary hypoparathyroidism were female gender, age < 50 years, BMI > 30 and previous bilateral thyroid or parathyroid surgery; former data gave similar results [18,38,48,49]. Lymphadenectomy, which is often mentioned to be a risk factor for postoperative hypoparathyroidism [50,51], showed no significant influence.

Although IONM is not recommended by the German [6] nor other guidelines [3,27], it was used in almost every procedure (98.9%). It is of note that intermittent IONM (77.4%) was significantly more often used than continuous IONM (21.9%), although continuous IONM is potentially better to avoid nerve injuries by traction compared to intermittent IONM. Continuous IONM is able to detect early progressive decrease in amplitudes and increase of latency, especially during traction, so that the surgeon can react immediately to signal changes [52]. Even in case of a loss of signal they can promptly respond to the adverse event [53]. In the largest retrospective study comparing C-IONM (2034 NAR) with I-IONM (850 NAR) the rate of transient vocal cord palsy was reduced in the C-IONM group by about 38% from 4.0% (34/850) to 2.9% (34/1184) [54]. There exists only one retrospective series of 209 patients comparing intermittent, continuous IONM and visualization alone with thyroidectomy for GD. RLN palsy occurred at a rate of 3.6% in the I-IONM group, 2.7% in the C-IONM group and 5.4% in the visualization group, and could not demonstrate a significant reduction of RLNP (*p* = 0.058) [55]. The presented analysis showed a rate of transient RLN palsy after use of continuous IONM of 3.4% compared to 4.1% when intermittent IONM was used (*p* = 0.059). Thus, the present StuDoQ data support the hypothesis that continuous IONM might be superior to intermittent IONM to prevent transient RLN palsy in GD. This hypothesis has to evaluated in the planned multicenter randomized controlled CITY trial.

The rate of reoperations due to postoperative bleeding after thyroid surgery for GD was 1.3%, which is in the range present in the literature (0.5 to 6.8%) [11,14,32].

A recent systematic review involving 10,743 patients with GD and 3336 non-GD patients with hyperthyroidism showed that the pooled prevalence of incidental thyroid carcinoma was 7.0% [51], which is comparable to the incidence of coincidental thyroid carcinoma of about 10% in patients who underwent thyroid surgery for any kind of benign goiter [56,57,58,59,60]. The presented series of 1808 cases showed a much lower risk with an incidental thyroid carcinoma prevalence of only 0.05%. A 10-fold increased thyroid cancer risk in GD patients within 3 years as reported in a population-based Taiwanese study in 2013 could not be analyzed, since the follow-up of the Thyroid/StudoQ registry is currently too short [61].

The present study has some drawbacks and some strengths. The analysis has the limitation of being a retrospective study, although the data are prospectively documented, externally monitored by independent surgeons and the respective departments that control of the participating centers. The 6-month follow-up data regarding RLNP and postoperative hypoparathyroidism were not available for about half of the patients, so that at present no definitive numbers can be given for permanent RLNP and postoperative hypoparathyroidism. However, the present study based on the StuDoQ/Thyroid registry gives a clear picture of the actual trends of thyroid surgery for GD in Germany, since about 20% of operated patients with GD in Germany were documented in 25 months.

## 5. Conclusions

Total thyroidectomy with IONM is the current most common surgical therapy for GD in Germany. The procedure is safe; however, postoperative hypoparathyroidism is the major complication, thus requiring more awareness.

## Figures and Tables

**Table 1:** Number of operations for Graves’ disease of contributing institutions and corresponding complication rates.

No. of Operations for GD/Evaluation Period	No. of Institutions *n* = 78	No. of Patients with GD *n* = 1808	Postoperative Hypoparathyroidism *n* = 525	Postoperative RLNP (per NAR)	Re-Operations Due to Bleeding
<10	38 (49%)	155 (8.6%)	72 (46.5%)	15/279 (5.4%)	1 (0.6%)
11–30	22 (28%)	411 (22.7%)	143 (34.8%)	42/772 (5.4%)	5 (1.2%)
31–50	7 (9%)	280 (15.5%)	62 (22.1%)	26/533 (4.9%)	3 (1.1%)
>51	11 (14%)	962 (53.2%)	248 (25.8%)	55/1845 (3.0%)	14 (1.5%)

The influence of center volume (cut-off > 30 operations for GD during the study period of 25 months) showed a significant reduction of hypoparathyroidism (*p* < 0.0005) and RLNP (*p* = 0.0229). The rate of reoperations due to bleeding was not influenced by the center volume. GD, Graves’ disease; RLNP, recurrent laryngeal nerve palsy; NAR, nerves at risk.

**Table 2:** Preoperative data of 1808 patients with GD (Graves’ disease) who underwent thyroid resection.

Parameter	% (*n*)
female	81.7% (1478)
age < 18 years	2.4% (43)
age > 70 years	3.5% (64)
BMI > 35	7.2% (131)
ASA 1/2	89.3% (1615)
Endocrine orbitopathy	35.7% (645)
Biochemical hyperthyroidism at operation	55.3% (999)
Thyrotoxic crisis at operation	0.3% (6)
Preoperative vocal cord assessment	98.5% (1780)
Preoperative VCP	0.3% (6)
Previous neck surgery	3.8% (68)

BMI—body mass index; ASA—American Society of Anesthesiologists; VCP—vocal cord palsy.

**Table 3:** Initial thyroid resections performed in 1808 patients with GD.

Type of Surgery	Number (%)
Total thyroidectomy	1668 (92.3%)
Hemithyroidectomy	98 (5.4%)
Hemithyroidectomy, contralateral node excision	4 (0.2%)
Hemithyroidectomy, contralateral subtotal resection	14 (0.8%)
Unilateral subtotal resection	3 (0.2%)
Bilateral subtotal resection	10 (0.6%)
Other resections (e.g., isthmus resection, node excision)	11 (0.6%)
All procedures	1808 (100%)

**Table 4:** Use of IONM (intraoperative neuromonitoring) and the rate of RLNP (recurrent laryngeal nerve palsy) per NAR (nerves at risk) in patients with GD.

Parameter	Number of NAR (%)	Unilateral RLNP * per NAR	Bilateral RLNP * per NAR	Postoperative not Examined
NAR	3429 * (100%)	134 (3.9%)	4 (0.1%)	97 (2.8%)
only visualization RLN	16 (0.5%)	0 (0%)	0 (0%)	0 (0%)
IONM	3409 (99.4%)	134 (3.9%)	4 (0.1%)	95 (2.8%)
intermittent IONM	2656 (77.4%)	108 (4.1%)	4 (0.1%)	72 (2.7%)
continuous IONM	753 (22.0%)	26 (3.4%)	0 (0%)	23 (3.1%)
no visualization, no IONM	4 (0.1%)	0 (0%)	0 (0%)	2 (50%)

* The procedures carried out during the evaluation period included 3489 nerves at risk, the intraoperative use of neuromonitoring or visualization was documented in 3429 nerves at risk. A postoperative laryngoscopy was done in 3332 nerves at risk, in 97 nerves at risk the postoperative examination of the vocal cord function was omitted. NAR—Nerves at risk; RLN—recurrent laryngeal nerve; IONM—intraoperative neuromonitoring; RLNP—recurrent laryngeal nerve palsy; vocal cord immobility was defined as palsy; impaired but notable vocal cord movement was defined as incomplete palsy. Both conditions were counted as impairment of RLN function.

**Table 5:** Transient postoperative hypoparathyroidism with regard to identification and autotransplantation of parathyroid glands.

Parameter	Number of Patients	Number of Patients with Transient Postoperative Hypoparathyroidism *	*p*-Value
All patients	1808 (100%)	525 (29.0%)	
no PG identified	31 (1.7%)	8 (25.8%)	
any PG identified	1777 (98.3%)	517 (29.1%)	0.69
1 PG identified	30 (1.6%)	5 (16.6%)	
2 or more PGs identified	1747 (96.6%)	512 (29.3%)	
no PG autotransplanted	1433 (79.3%)	362 (25.2%)	<0.0001
any PG autotransplanted	375 (20.7%)	163 (43.5%)	
1 PG autotransplanted	3 (0.1%)	3 (100%)	
2 PG autotransplanted	327 (18.1%)	131 (40.1%)	
1 or 2 PG autotransplanted	330 (18.3%)	134 (40.6%)	
>2 PG autotransplanted	45 (2.5%)	29 (64.4%)	

PG—parathyroid gland; *—postoperative hypoparathyroidism was defined at intact PTH < 15 pg/L and serum calcium at <2.1 mmol/L with the necessity of Ca—substitution at discharge.

**Table 6:** Uni- and multivariate logistic regression of parameters associated with postoperative temporary hypoparathyroidism.

Parameter	Univariate Analysis		Multivariate Analysis	
	OR (95% CI)	*p*-Value	OR (95% CI)	*p*-Value
Case load GD/study period > 30	0.543 (0.439–0.672)	<0.0005	0.490 (0.393–0.611)	<0.0005
Case load thyroid resection/year > 301	1.759 (1.351–2.290)	<0.0005	0.501 (0.392–0.640)	<0.0005
Age > 50 years	0.660 (0.531–0.819)	<0.0005	0.690 (0.552–0.864)	0.001
BMI > 30	0.701 (0.520–0.944)	0.019	1.349 (1.064–1.711)	0.014
Male gender	0.580 (0.434–0.774)	<0.0005	0.666 (0.494–0.897)	0.007
Previous bilateral thyroid/parathyroid surgery	0.310 (0.109–0.883)	0.028	0.330 (0.114–0.959)	0.042
Autotransplantation PG	2.275 (1.796–2.881)	<0.0005	2.276 (1.786–2.902)	<0.0005
Surgical method minimally invasive	1.370 (0.547–3.430)	0.502		
Previous neck surgery overall	0.683 (0.381–1.222)	0.199		
Lymphadenectomy	0.620 (0.375–1.02)	0.063		
Thyroid volume > 100 g	1.170 (0.689–1.985)	0.080		
Preoperative TSH level	0.913 (0.737–1.131)	0.405		
Preoperative PTH level	0.848 (0.496–1.451)	0.548		

BMI—body mass index; PG—parathyroid gland.

## References

[1] Liu Z.W., Masterson L., Fish B., Jani P., Chatterjee K. (2015). Thyroid surgery for Graves’ disease and Graves’ ophthalmopathy. Cochrane Database Syst. Rev..

[2] Burch H.B., Cooper D.S. (2015). Management of graves disease a review. JAMA.

[3] Ross D.S., Burch H.B., Cooper D.S., Greenlee M.C., Laurberg P., Maia A.L., Rivkees S.A., Samuels M., Sosa J.A., Stan M.N. (2016). 2016 American Thyroid Association Guidelines for Diagnosis and Management of Hyperthyroidism and Other Causes of Thyrotoxicosis. Thyroid.

[4] Dietlein M., Grünwald F., Schmidt M., Schneider P., Verburg F., Luster M. (2015). DGN-Handlungsempfehlung (S1-Leitlinie) Radioiodtherapie bei Benignen Schilddrüsenerkr (Version 5) Stand: 10/2015—AWMF-Registernummer: 031-003. https://www.awmf.org/uploads/tx_szleitlinien/031-003l_S1_Radioiodtherapie_benigne_Schilddruesenerkrankungen_2015-10-abgelaufen.pdf.

[5] Genovese B.M., Noureldine S.I., Gleeson E.M., Tufano R.P., Kandil E. (2013). What is the best definitive treatment for graves’ disease? A systematic review of the existing literature. Ann. Surg. Oncol..

[6] Bartsch D.K., Clerici T., Dotzenrath C., Dralle H., Goretzki P.E., Hermann M., Holzer K., Kußmann J., Lorenz K., Nies C. (2015). Leitlinie der Deutschen Gesellschaft für Allgemein- und Viszeralchirurgie—Chirurgische Arbeitsgemeinschaft Endokrinologie: Operative Therapie Benigner Schilddrüsenerkrankungen. AWMF/11/003/0021. https://www.awmf.org/uploads/tx_szleitlinien/088-007l_S2k_operative_Therapie_benigner_Schilddrüsenerkrankungen_2015-10-abgelaufen_01.pdf.

[7] Palit T.K., Miller C.C., Miltenburg D.M. (2000). The efficacy of thyroidectomy for Graves’ disease: A meta-analysis. J. Surg. Res..

[8] Werga-Kjellman P., Zedenius J., Tallstedt L., Träisk F., Lundell G., Wallin G. (2001). Surgical treatment of hyperthyroidism: A ten-year experience. Thyroid.

[9] Guo Z., Yu P., Liu Z., Si Y., Jin M. (2013). Total thyroidectomy vs. bilateral subtotal thyroidectomy in patients with Graves’ diseases: A meta-analysis of randomized clinical trials. Clin. Endocrinol..

[10] Papanastasiou A., Sapalidis K., Goulis D.G., Michalopulos N., Mareti E., Mantalovas S., Kesisoglou I. (2019). Thyroid nodules as a risk factor for thyroid cancer in patients with Grave’s disease: A systematic review and meta-analysis of observational studies in surgically treated patients. Clin. Endocrinol..

[11] Witte J., Goretzki P.E., Dotzenrath C., Simon D., Felis D., Neubauer M., Röher H.D. (2000). Surgery for Graves’ disease: Total versus subtotal thyroidectomy—Results of a prospective randomized trial. World J. Surg..

[12] Järhult J., Rudberg C., Larsson E., Selvander H., Sjövall K., Winsa B., Rastad J., Karlsson F.A. (2005). TEO Study Group Graves’ disease with moderate-severe endocrine ophthalmopathy-long term results of a prospective, randomized study of total or subtotal thyroid resection. Thyroid.

[13] Barczyński M., Konturek A., Hubalewska-Dydejczyk A., Gołkowski F., Nowak W. (2012). Randomized clinical trial of bilateral subtotal thyroidectomy versus total thyroidectomy for Graves’ disease with a 5-year follow-up. Br. J. Surg..

[14] Maurer E., Maschuw K., Reuss A., Zieren H.U., Zielke A., Goretzki P., Simon D., Dotzenrath C., Steinmüller T., Jähne J. (2019). Total Versus Near-Total Thyroidectomy in Graves Disease. Ann. Surg..

[15] Kwon H., Kim J kyu Lim W., Moon B.I., Paik N.S. (2019). Increased risk of postoperative complications after total thyroidectomy with Graves’ disease. Head Neck..

[16] Thomusch O., Sekulla C., Billmann F., Seifert G., Dralle H., Lorenz K., PETS2 Study Group (2018). Risk profile analysis and complications after surgery for autoimmune thyroid disease. Br. J. Surg..

[17] Feroci F., Rettori M., Borrelli A., Coppola A., Castagnoli A., Perigli G., Cianchi F., Scatizzi M. (2014). A systematic review and meta-analysis of total thyroidectomy versus bilateral subtotal thyroidectomy for Graves’ disease. Surgery.

[18] Thomusch O., Machens A., Sekulla C., Ukkat J., Brauckhoff M., Dralle H. (2003). The impact of surgical technique on postoperative hypoparathyroidism in bilateral thyroid surgery: A multivariate analysis of 5846 consecutive patients. Surgery.

[19] Erbil Y., Barbaros U., Ozbey N., Aral F., Özarmaǧan S. (2009). Risk factors of incidental parathyroidectomy after thyroidectomy for benign thyroid disorders. Int. J. Surg..

[20] Musholt T.J., Clerici T., Frilling A., Goretzki P.E., Hermann M., Kußmann J., Lorenz K., Niederle B., Nies C., Simon D. (2012). Leitlinie der Deutschen Gesellschaft für Allgemein- und Viszeralchirurgie—Chirurgische Arbeitsgemeinschaft Endokrinologie: Operative Therapie Maligner Schilddrüsenerkrankungen. AWMF-Leitlinien-Register Nr. 088/022. https://www.awmf.org/leitlinien/detail/anmeldung/1/ll/031-056OL.html.

[21] Bartsch D., Dotzenrath C., Vorländer C., Zielke A., Weber T., Buhr H.J., Klinger C., Lorenz K., StuDoQ/Thyroid Study Group (2019). Current Practice of Surgery for Benign Goitre—An Analysis of the Prospective DGAV StuDoQ|Thyroid Registry. J. Clin. Med..

[22] Rubio G.A., Koru-Sengul T., Vaghaiwalla T.M., Parikh P.P., Farra J.C., Lew J.I. (2017). Postoperative outcomes in graves’ disease patients: Results from the nationwide inpatient sample database. Thyroid.

[23] Alesina P.F., Singaporewalla R.M., Eckstein A., Lahner H., Walz M.K. (2011). Is minimally invasive, video-assisted thyroidectomy feasible in Graves’ disease?. Surgery.

[24] Kwon H., Yi J.W., Song R.Y., Chai Y.J., Kim S., Choi J.Y., Lee K.E. (2016). Comparison of Bilateral Axillo-Breast Approach Robotic Thyroidectomy with Open Thyroidectomy for Graves’ Disease. World J. Surg..

[25] Park J.H., Lee C.R., Park S., Jeong J.S., Kang S.W., Jeong J.J., Nam K.H., Chung W.Y., Park C.S. (2013). Initial experience with robotic gasless transaxillary thyroidectomy for the management of graves disease: Comparison of conventional open versus robotic thyroidectomy. Surg. Laparosc. Endosc. Percutaneous Tech..

[26] Garstka M., Kandil E., Saparova L., Bechara M., Green R., Haddad A.B., Kang S.W., Aidan P. (2018). Surgery for Graves’ disease in the era of robotic-assisted surgery: A study of safety and feasibility in the Western population. Langenbeck Arch. Surg..

[27] Kahaly G.J., Bartalena L., Hegedüs L., Leenhardt L., Poppe K., Pearce S.H. (2018). 2018 European thyroid association guideline for the management of graves’ hyperthyroidism. Eur. Thyroid J..

[28] Musholt T.J., Bockisch A., Clerici T., Dotzenrath C., Dralle H., Goretzki P.E., Hermann M., Holzer K., Karges W., Krude H. (2018). Update of the S2k guidelines: Surgical treatment of benign thyroid diseases. Chirurg.

[29] Cipolla C., Graceffa G., Calamia S., Fiorentino E., Pantuso G., Vieni S., Latteri M. (2019). The value of total thyroidectomy as the definitive treatment for Graves’ disease: A single centre experience of 594 cases. J. Clin. Transl. Endocrinol..

[30] Lin Y.S., Lin J.D., Hsu C.C., Yu M.C. (2017). The long-term outcomes of thyroid function after subtotal thyroidectomy for Graves’ hyperthyroidism. J. Surg. Res..

[31] Limonard E.J., Bisschop P.H., Fliers E., Van Dijkum E.J.N. (2012). Thyroid function after subtotal thyroidectomy in patients with Graves’ hyperthyroidism. Sci. World J..

[32] Chi S.Y., Hsei K.C., Sheen-Chen S.M., Chou F.F. (2005). A prospective randomized comparison of bilateral subtotal thyroidectomy versus unilateral total and contralateral subtotal thyroidectomy for Graves’ disease. World J. Surg..

[33] Hallgrimsson P., Nordenström E., Almquist M., Bergenfelz A.O.J. (2012). Risk factors for medically treated hypocalcemia after surgery for Graves’ disease: A swedish multicenter study of 1,157 patients. World J. Surg..

[34] Wilhelm S.M., McHenry C.R. (2010). Total thyroidectomy is superior to subtotal thyroidectomy for management of graves’ disease in the United States. World J. Surg..

[35] Al Qubaisi M., Haigh P.I. (2019). Hypocalcemia after Total Thyroidectomy in Graves Disease. Perm. J..

[36] Ebrahimi H., Edhouse P., Lundgren C.I., McMullen T., Sidhu S., Sywak M., Delbridge L. (2009). Does autoimmune thyroid disease affect parathyroid autotransplantation and survival?. Anz. J. Surg..

[37] Palazzo F.F., Sywak M.S., Sidhu S.B., Barraclough B.H., Delbridge L.W. (2005). Parathyroid autotransplantation during total thyroidectomy—Does the number of glands transplanted affect outcome?. World J. Surg..

[38] Edafe O., Antakia R., Laskar N., Uttley L., Balasubramanian S.P. (2014). Systematic review and meta-analysis of predictors of post-thyroidectomy hypocalcaemia. Br. J. Surg..

[39] Karakas E., Osei-Agyemang T., Schlosser K., Hoffmann S., Zielke A., Rothmund M., Hassan I. (2008). The impact of parathyroid gland autotransplantation during bilateral thyroid surgery for graves’ disease on postoperative hypocalcaemia. Endocr. Regul..

[40] Lin D.T., Patel S.G., Shaha A.R., Singh B., Shah J.P. (2002). Incidence of inadvertent parathyroid removal during thyroidectomy. Laryngoscope.

[41] Sippel R.S., Özgül Ö., Hartig G.K., Mack E.A., Chen H. (2007). Risks and consequences of incidental parathyroidectomy during thyroid resection. Anz. J. Surg..

[42] Page C., Strunski V. (2007). Parathyroid risk in total thyroidectomy for bilateral, benign, multinodular goitre: Report of 351 surgical cases. J. Laryngol. Otol..

[43] Lavazza M., Liu X., Wu C., Anuwong A., Kim H.Y., Liu R., Randolph G.W., Inversini D., Boni L., Rausei S. (2016). Indocyanine green-enhanced fluorescence for assessing parathyroid perfusion during thyroidectomy. Gland Surg..

[44] Lang B.H.H., Wong C.K.H., Hung H.T., Wong K.P., Mak K.L., Au K.B. (2017). Indocyanine green fluorescence angiography for quantitative evaluation of in situ parathyroid gland perfusion and function after total thyroidectomy. Surgery.

[45] McWade M.A., Sanders M.E., Broome J.T., Solórzano C.C., Mahadevan-Jansen A. (2016). Establishing the clinical utility of autofluorescence spectroscopy for parathyroid detection. Surgery.

[46] Ladurner R., Al Arabi N., Guendogar U., Hallfeldt K.K.J., Stepp H., Gallwas J.K.S. (2018). Near-infrared autofluorescence imaging to detect parathyroid glands in thyroid surgery. Ann. R. Coll. Surg. Engl..

[47] De Leeuw F., Breuskin I., Abbaci M., Casiraghi O., Mirghani H., Lakhdar A.B., Laplace-Builhe C., Hartl D. (2016). Intraoperative Near-infrared Imaging for Parathyroid Gland Identification by Auto-fluorescence: A Feasibility Study. World J. Surg..

[48] Bergenfelz A., Jansson S., Kristoffersson A., Mårtensson H., Reihnér E., Wallin G., Lausen I. (2008). Gender Complications to thyroid surgery: Results as reported in a database from a multicenter audit comprising 3,660 patients. Langenbecks Arch Surg..

[49] Cappellani A., Di Vita M., Zanghì A., Lo Menzo E., Cavallaro A., Alfano G., Giuffrida D. (2008). The recurrent goiter: Prevention and management. Ann. Ital. Chir..

[50] Orloff L.A., Wiseman S.M., Bernet V.J., Fahey T.J., Shaha A.R., Shindo M.L., Snyder S.K., Stack B.C., Sunwoo J.B., Wang M.B. (2018). American Zhyroid Association Statement on Postoperative Hypoparathyroidism: Diagnosis, Prevention and management in Adults. Thyroid.

[51] Antakia R., Edafe O., Uttley L., Balasubramanian S.P. (2015). Effectiveness of preventative and other surgical measures on hypocalcemia following bilateral thyroid surgery: A systematic review and meta-analysis. Thyroid.

[52] Kandil E., Mohsin K., Murcy M.A., Randolph G.W. (2018). Continuous vagal monitoring value in prevention of vocal cord paralysis following thyroid surgery. Laryngoscope.

[53] Dionigi G., Donatini G., Boni L., Rausei S., Rovera F., Tanda M.L., Kim H.Y., Chiang F.Y., Wu C.W., Mangano A. (2013). Continuous monitoring of the recurrent laryngeal nerve in thyroid surgery: A critical appraisal. Int. J. Surg..

[54] Jonas J., Boskovic A. (2014). Intraoperative neuromonitoring (IONM) for recurrent laryngeal nerve protection: Comparison of intermittent and continuous nerve stimulation. Surg. Technol. Int..

[55] Zhou L., Dionigi G., Pontin A., Pino A., Caruso E., Wu C.W., Sun H., Tufano R.P., Kim H.Y. (2019). How does neural monitoring help during thyroid sugery for Graves’ disease?. J. Clin. Transl. Endocrinol..

[56] Jia Q., Li X., Liu Y., Ling L., Kwong J.S., Ren K., Jiang Y., Sun X., Tian H., Li S. (2018). Incidental thyroid carcinoma in surgery-treated hyperthyroid patients with Graves’ disease: A systematic review and meta-analysis of cohort studies. Cancer Manag. Res..

[57] Fama F., Sindoni A., Cicciu M., Polito F., Piquard A., Saint-Marc O., Gioffre-Florio M., Benvenga S. (2018). Preoperatively undiagnosed papillary thyroid carcinoma in patients thyroidectomized for benign multinodular goiter. Arch. Endocrinol. Metab..

[58] Miccoli P., Minuto M.N., Galleri D., D’Agostino J., Basolo F., Antonangeli L., Aghini-Lombardi F., Berti P. (2006). Incidental thyroid carcinoma in a large series of consecutive patients operated on for benign thyroid disease. Anz. J. Surg..

[59] Pezzolla A., Marzaioli R., Lattarulo S., Docimo G., Conzo G., Ciampolillo A., Barile G., Anelli F.M., Madaro A. (2014). Incidental carcinoma of the thyroid. Int. J. Surg..

[60] Botrugno I., Lovisetto F., Cobianchi L., Zonta S., Klersy C., Vailati A., Dionigi P., Jemos V. (2011). Incidental carcinoma in multinodular goiter: Risk factors. Am. Surg..

[61] Chen Y.K., Lin C.L., Chang Y.J., Cheng F.T., Peng C.L., Sung F.C., Cheng Y.H., Kao C.H. (2013). Cancer risk in patients with Graves’ disease: A nationwide cohort study. Thyroid.

